# Urinary Galectin-3 as a Novel Biomarker for the Prediction of Renal Fibrosis and Kidney Disease Progression

**DOI:** 10.3390/biomedicines10030585

**Published:** 2022-03-02

**Authors:** Shuo-Ming Ou, Ming-Tsun Tsai, Huan-Yuan Chen, Fu-An Li, Kuo-Hua Lee, Wei-Cheng Tseng, Fu-Pang Chang, Yao-Ping Lin, Ruey-Bing Yang, Der-Cherng Tarng

**Affiliations:** 1 Division of Nephrology, Department of Medicine, Taipei Veterans General Hospital, Taipei 112201, Taiwan; okokyytt@gmail.com (S.-M.O.); mingtsun74@gmail.com (M.-T.T.); dadabim3520@gmail.com (K.-H.L.); wctseng@gmail.com (W.-C.T.); linyp@vghtpe.gov.tw (Y.-P.L.); 2 School of Medicine, National Yang Ming Chiao Tung University, Taipei 112304, Taiwan; 3 Institute of Clinical Medicine, National Yang Ming Chiao Tung University, Taipei 112304, Taiwan; 4 Center for Intelligent Drug Systems and Smart Bio-Devices (IDS2B), National Yang Ming Chiao Tung University, Hsinchu 300093, Taiwan; 5 Institute of Biomedical Sciences, Academia Sinica, Taipei 115201, Taiwan; hchen9@ibms.sinica.edu.tw (H.-Y.C.); falee@ibms.sinica.edu.tw (F.-A.L.); 6 Department of Pathology and Laboratory Medicine, Taipei Veterans General Hospital, Taipei 112201, Taiwan; fpchang@vghtpe.gov.tw; 7 Inflammation and Immunity Research Center, National Yang Ming Chiao Tung University, Taipei 112304, Taiwan; 8 Department and Institute of Physiology, National Yang Ming Chiao Tung University, Taipei 112304, Taiwan

**Keywords:** galectin-3, kidney disease progression, renal biopsy, renal fibrosis, urinary biomarkers

## Abstract

Plasma galectin-3 (Gal-3) is associated with organ fibrosis, but whether urinary Gal-3 is a potential biomarker of kidney disease progression has never been explored. Between 2018 and 2021, we prospectively enrolled 280 patients who underwent renal biopsy and were divided into three groups based on their urinary Gal-3 levels (<354.6, 354.6–510.7, and ≥510.8 pg/mL) to assess kidney disease progression (defined as ≥40% decline in the estimated glomerular filtration rate or end-stage renal disease) and renal histology findings. Patients in the highest urinary Gal-3 tertile had the lowest eGFRs and highest proteinuria levels. In multivariate Cox regression models, patients in the highest tertile had the highest risk of kidney disease progression (adjusted hazard ratio, 4.60; 95% confidence interval, 2.85–7.71) compared to those in the lowest tertile. Higher urinary Gal-3 levels were associated with more severe renal fibrosis. Intrarenal mRNA expression of LGALS3 (Gal-3-encoded gene) was most correlated with the renal stress biomarkers (IGFBP7 and TIMB2), renal function biomarkers (PTGDS) and fibrosis-associated genes (TGFB1). The urinary Gal-3 level may be useful for the identification of patients at high risk of kidney disease progression and renal fibrosis, and for the early initiation of treatments for these patients.

## 1. Introduction

Chronic kidney disease (CKD) constitutes a global health burden with a rising incidence and prevalence [1]. It may cause renal function decline that progresses to end-stage renal disease (ESRD) [2]. The early identification of patients at greater risk of such decline would allow the prompt initiation of interventions or treatment [3]. As it can be collected non-invasively and is easily accessible, urine has become an important and useful source of disease biomarkers, especially for early-stage disease [4,5]. Proteinuria is the most reported urinary biomarker of renal function decline, but it may not be ideal for the early detection of this condition because it is accompanied by evident renal damage and lacks sensitivity [6].

Galectin-3 (Gal-3) binds to β-galactoside sugars in its carbohydrate recognition domain to exert diverse properties, including cell adhesion and proliferation via several glycosylated matrix proteins (i.e., laminin, fibronectin, and integrins) [7,8]. Gal-3 also contributes to pathological processes such as inflammation, angiogenesis, and organ fibrogenesis in the presence of tissue injury [8,9,10]. The depletion of Gal-3 was found to reduce collagen matrix accumulation and the severity of fibrosis [11,12]. Previous epidemiological studies have shown that higher plasma or serum Gal-3 levels were associated with increased risks of incident CKD and rapid renal function decline, even with accounting for known clinical CKD predictors [13,14,15]. In another study, higher plasma Gal-3 levels were found to be associated with more severe renal fibrosis in the findings of kidney biopsy [16].

Research on Gal-3 levels, however, has been conducted with blood, rather than urine. The aim of this study was to examine whether the urinary Gal-3 level is also a useful biomarker enabling the accurate prediction of the risk of kidney disease progression, which could help clinicians to early initiate renoprotective strategies to slow such progression. In addition, we examined correlations between the urinary Gal-3 level and histopathological findings of kidney biopsy. Finally, we explored associations of intrarenal mRNA expression of Gal-3-encoded gene LGALS3 with the gene expressions of known CKD biomarkers and fibrosis-associated genes in kidney biopsy specimens.

## 2. Materials and Methods

### 2.1. Study Design and Populations

We prospectively enrolled patients at Taipei Veterans General Hospital, a tertiary medical center, who underwent renal biopsies between 2018 and 2021. Exclusion criteria were age < 20 years, lack of urinary Gal-3 measurement, and unwillingness to participate in the study or provide written informed consent.

### 2.2. Clinical Variables

Our analyses involved the following clinical variables: age, sex, estimated glomerular filtration rate (eGFR), spot urinary protein-to-creatinine ratio (UPCR), uric acid, albumin, alanine transaminase, hypertension, dyslipidemia, diabetes mellitus, systemic lupus erythematosus, coronary artery disease, congestive heart failure, stroke, chronic obstructive pulmonary disease, peptic ulcer disease, and malignancy. The eGFR was calculated using the Chronic Kidney Disease Epidemiology Collaboration equation [17]. Kidney disease progression was defined as ≥40% decline in the eGFR from baseline or ESRD (i.e., the initiation of hemodialysis or peritoneal dialysis, the receipt of a kidney transplant or eGFR < 15 mL/min/1.73 m^2^) [18,19]. Patients were followed until either death occurred or the study period ended, whichever occurred first.

### 2.3. Blood and Urine Sampling and Gal-3 Measurement

Blood and spot urine samples collected at the time of kidney biopsy were centrifuged for 15 min at 2000× *g* and 4 °C before being stored at −80 °C. These samples were thawed to 4 °C before further analysis. The plasma and urinary Gal-3 levels were measured in picograms per milliliter using an in-house multiplex bead-based immunoassay, as described previously [20,21].

### 2.4. Kidney Biopsy Specimen Analysis

The histopathological findings of the kidney biopsy specimens were reviewed with light microscopy, immunofluorescence staining, and electron microscopy by an experienced pathologist. For light microscopic analysis, the specimens were stained with hematoxylin and eosin, periodic acid–Schiff, and Masson’s trichrome stains. Immunofluorescence was used to examine immunoglobulin and complement deposits, and transmission electron microscopy was used to examine the ultrastructure of the glomerulus and tubulointerstitium. Detailed findings from these analyses were also examined in relation to the urinary Gal-3 level for different kidney disease etiologies. The collected kidney biopsy specimens were immediately frozen in −80 °C with RNAlater reagent. Using a manual microdissection technique, the tissue samples were divided into glomerular compartments and tubulointerstitial compartments. RNA of the dissected tubulointerstitial compartments were isolated, followed by complementary deoxyribonucleic acid synthesis. The renal tubulointerstitial compartments were further analyzed to explore the associations between intrarenal mRNA expression of LGALS3 (the Gal-3 gene) and gene expressions of other known CKD biomarkers or fibrosis-associated genes as follows: genes of renal stress biomarkers (IGFBP7 and TIMP2), renal function biomarkers (PTGDS and B2M), tubular injury biomarkers (HAVCR1, LCN2, and PTX3), inflammation biomarkers (IL6 and GDF15), and fibrosis-associated genes (TGFB1, COL1A1,ACTA2, DES and EGF) [22,23,24,25,26]. Relative expression of mRNA levels was normalized to the housekeeping gene GAPDH mRNA levels.

### 2.5. Statistical Analysis

Categorical data are presented as number and percentages and analyzed using chi-squared test. Continuous data are presented as medians with interquartile ranges and analyzed using one-way ANOVA. The Cox proportional-hazards model was used to assess the risk of kidney disease progression according to tertiles of the urinary Gal-3 level. Kaplan–Meier curves were used to estimate the probability of kidney disease progression and the tertiles of urinary Gal-3 level. Recently, net reclassification (NRI) and integrated discrimination improvement (IDI) have been introduced to measure the improvement in model prediction based on the addition of a new biomarker to the existing prediction model. In our study, we calculated the NRI and IDI to examine the net effects of adding plasma or urinary Gal-3 for predicting kidney disease progression [27,28]. In addition, the Akaike Information Criterion (AIC) and Bayesian Information Criterion (BIC) values were calculated to estimate the quality of the models, and adjusted R-squared values were also calculated [29,30]. To determine the magnitude of correlations between the intrarenal mRNA expressions of LGALS3 and gene expressions of known CKD biomarkers and fibrosis-associated genes in the kidney biopsy specimens, we generated correlation plots. The data were analyzed using SAS software (version 9.4; SAS Institute Inc., Cary, NC, USA) and R software (version 3.5.2 for Windows; R Core Team, Vienna, Austria). *p* values < 0.05 were considered to be significant.

## 3. Results

### 3.1. Baseline Characteristics of Participants

Table 1 shows the demographic and clinical characteristics of the study participants. In total, 280 patients (60.7% male) with a mean (standard deviation (SD)) age of 56.2 (16.5) years were further stratified by tertiles of the urinary Gal-3 level (<354.6, 354.6–510.7, and ≥510.8 pg/mL). Seventy-three (26.1%) patients had diabetes mellitus and 115 (41.1%) patients had hypertension. To explore the relationship between urinary Gal-3 levels and renal function, the eGFR values showed significant decreases across urinary Gal-3 tertiles (*p* < 0.001), suggesting a worse renal function with higher urinary Gal-3 levels. Increased UPCR levels were observed across urinary Gal-3 tertiles (*p* < 0.001). Moreover, the proportion of participants with diabetes mellitus increased across urinary Gal-3 tertiles (*p* = 0.030).

### 3.2. Associations of the Urinary Gal-3 Level with Plasma Gal-3, eGFR, Creatinine and Proteinuria

**Figure 1:**
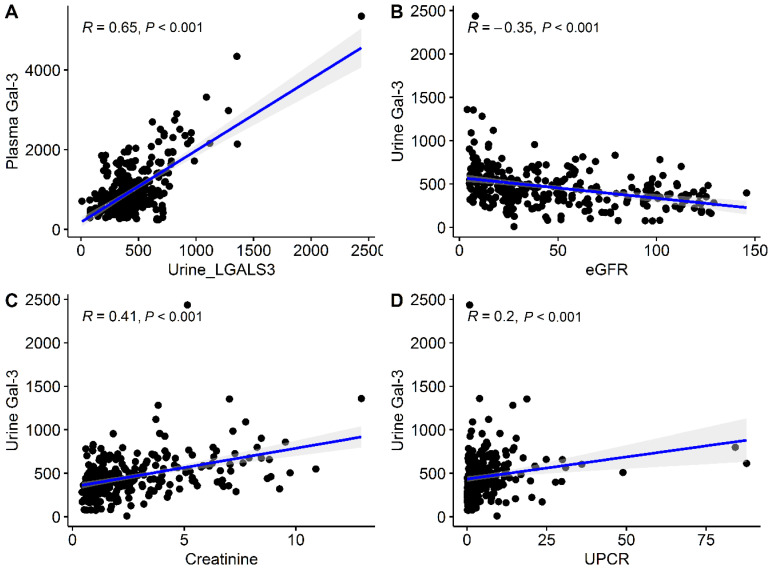
Correlation between urinary Gal-3, plasma Gal-3, eGFR, creatinine, and UPCR. (**A**) Correlation plots between urinary Gal-3 and plasma Gal-3 levels. (**B**) Correlation plots between urinary Gal-3 level and eGFR. (**C**) Correlation plots between urinary Gal-3 level and creatinine. (**D**) Correlation plots between urinary Gal-3 level and UPCR. *Abbreviations*: Gal-3, Galectin-3; eGFR, estimated glomerular filtration rate; UPCR, spot urine protein–creatinine ratio.

**Figure 2:**
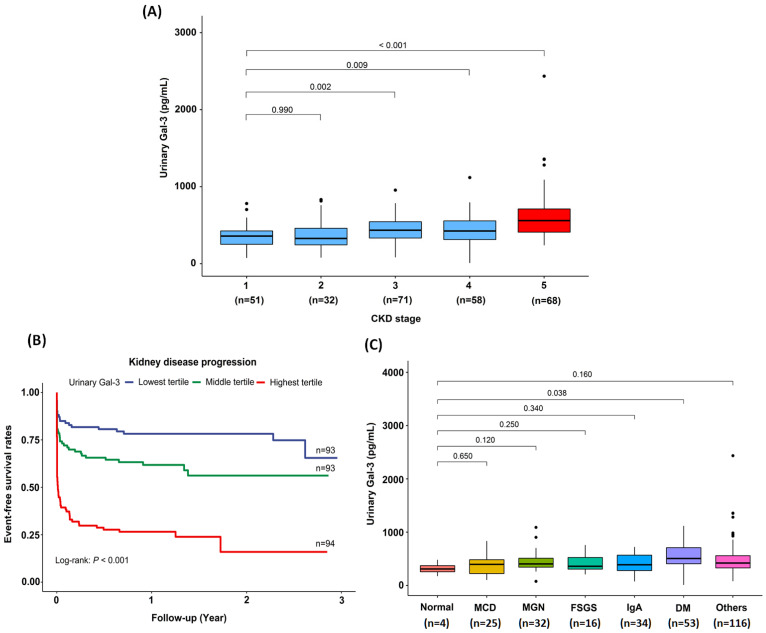
The associations between urinary Gal-3 levels and renal outcomes. (**A**) Urinary Gal-3 levels increased as CKD stage progressed. (**B**) Kaplan–Meier curves for the risks of kidney disease progression in tertiles of urinary Gal-3. (**C**) The associations between different pathological diagnoses and urinary Gal-3 levels. *Abbreviations*: Gal-3, Galectin-3; CKD, chronic kidney disease; MCD, minimal change disease; MGN, membranous glomerulonephritis; FSGS, focal segmental glomerulosclerosis; IgA, immunoglobulin A; DM, diabetes mellitus.

The urinary Gal-3 level correlated inversely with the eGFR (*r* = −0. 35, *p* < 0.001), and positively with plasma Gal-3 level (*r* = 0.65, *p* < 0.001), creatinine level (*r* = 0.41, *p* < 0.001) and UPCR (*r* = 0.20, *p* < 0.001; Figure 1). Interestingly, there was a gradual increase in urinary Gal-3 levels with CKD stage progression, with an increase that was greatest among patients with CKD stage 5 (Figure 2A).

### 3.3. Association of the Urinary Gal-3 Levels with Kidney Disease Progression

In multivariate Cox regression (Table 2), adjusted hazard ratios (aHRs) with 95% confidence intervals (CIs) for kidney disease progression among patients in the highest (aHR, 4.60; 95% CI, 2.85–7.71; *p* < 0.001) and middle (aHR, 1.82; 95% CI, 1.08–3.15; *p* = 0.027) urinary Gal-3 tertile relative to those in the lowest tertile. Each 100 pg/mL increase in the urinary Gal-3 levels increased the risks of kidney disease progression (aHR, 1.19; 95% CI, 1.13–1.25; *p* < 0.001). Kaplan–Meier analysis showed that patients in the highest urinary Gal-3 tertile were more likely to be at risk of kidney disease progression than were those in the middle and lowest tertiles (log-rank test, *p* < 0.001; Figure 2B).

### 3.4. NRI, IDI, AIC, BIC and Adjusted R^2^ for the Combined Assessment of eGFR and Gal-3 in Predicting Kidney Disease Progression

The NRI was 0.5395 (95% CI: 0.3133–0.7657; *p* < 0.001) and IDI was 0.0626 (95% CI: 0.0344–0.0907; *p* < 0.001) in model 2 compared to model 1, showing that the predictive performance was improved by adding urinary Gal-3 to the model (Table 3). Similarly, the NRI was 0.7333 (95% CI: 0.5172–0.9494; *p* < 0.001) and IDI was 0.0633 (95% CI: 0.0333–0.0934; *p* < 0.001) in model 3 compared to model 1, showing that the predictive performance was improved by adding plasma Gal-3 to the model. Of note, the IDI was increased to 0.090 in model 4 by adding both urinary and plasma Gal-3 to the model 1. The AIC and BIC values were reduced in model 2, model 3 and model 4 compared to model 1, suggesting that adding urinary Gal-3 or plasma Gal-3 or both provided better model fits.

### 3.5. Associations of the Urinary Gal-3 Level with the Histopathological Findings in Kidney Biopsy Specimens

The detailed associations of urinary Gal-3 levels and histopathological findings are shown in Table 4. Higher levels of urinary Gal-3 level were associated with more severe GBM double contour (*p* < 0.001), GBM rigid (*p* < 0.001), GBM thickening (*p* = 0.001), GBM collapse (*p* = 0.009), endocapillary hypertrophy (*p* < 0.001), interstitial inflammation (*p* = 0.003), interstitial fibrosis (*p* = 0.003), tubular atrophy (*p* = 0.002). The detailed pathological diagnoses of kidney biopsies in our study are provided in Table S1. Among different pathological diagnosis, patients with diabetic nephropathy had the highest urinary Gal-3 levels (Figure 2C). The associations between different pathological diagnoses and eGFR are shown in Figure S1.

### 3.6. Associations of the Intrarenal RNA Expression of LGALS3, Known CKD Biomarkers and Fibrosis-Associated Genes in Kidney Biopsy Specimens

**Figure 3:**
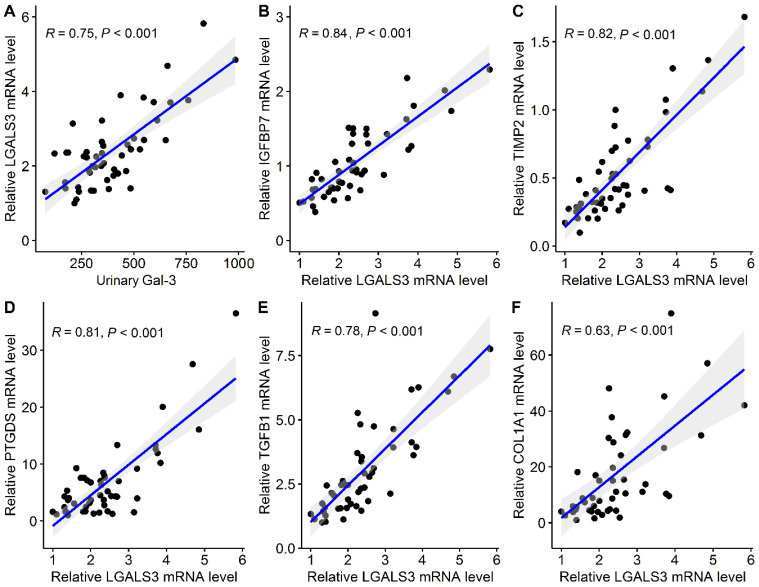
Correlation plots of (**A**) urinary Gal-3 and intrarenal LGALS3 as well as LGALS3 with (**B**) IGFBP7, (**C**) TIMP2, (**D**) PTGDS, (**E**) TGFB1 and (**F**) COL1A1 in kidney biopsy specimens. Abbreviations: Gal-3, galectin-3.

The intrarenal RNA expressions were examined in 50 patients who had undergone percutaneous kidney biopsy, and the detailed clinical characteristics of these patients are presented in Table S2. The urinary Gal-3 level correlated positive with intrarenal LGALS3 (*r* = 0.75, *p* < 0.001; Figure 3A). In the correlation plots, correlation coefficients of LGALS3 with genes of renal stress biomarkers, IGFBP7 and TIMP2 and renal function markers, PTGDS were 0.84, 0.82 and 0.81, respectively (Figure 3B–D). In addition, LGALS3 was most correlated with fibrosis-associated genes, TGFB1 (*r* = 0.78) and COL1A1 (*r* = 0.63), suggesting that LGALS3 is involved in transforming growth factor-β (TGF-β)-induced fibrotic processes (Figure 3E,F). The correlation coefficients of LGALS3 with other CKD biomarkers and fibrosis-associated genes are shown in Figure S2.

## 4. Discussion

The main aim of this study was to test the ability of the urinary Gal-3 level to predict kidney disease progression and the degrees of renal fibrosis in a cohort of patients who underwent renal biopsy. First, we found that urinary Gal-3 levels were inversely related to eGFR but were positively related to creatinine and UPCR. Secondly, we found that the highest urinary Gal-3 tertile was associated with increased risks of kidney disease progression compared to the lowest tertile. Thirdly, higher urinary Gal-3 levels were associated with more severe renal fibrosis. Combining urinary and plasma Gal-3 may provide better predictive performance for kidney disease progression. Finally, we observed a correlation between intrarenal RNA expression of LGALS3 and genes of renal stress biomarkers (IGFBP7 and TIMB2), renal function markers (PTGDS) and fibrosis-associated genes (TGFB1) in kidney biopsy specimens. Taken together, our data suggest that urinary Gal-3 may be a useful biomarker for the identification of individuals at risk of kidney disease progression and those predisposing to renal fibrosis, which may contribute to the pathogenesis of CKD early in the disease course.

To date, the prediction of kidney disease progression has been attempted by incorporating traditional laboratory data, such as creatinine, eGFR as well as other clinical and biochemical variables [31,32]. However, the individual course of kidney disease varies, and these laboratory data may not be able to predict progression accurately [33]. Serum and plasma Gal-3, which has been recently shown to be associated with incident CKD and eGFR decline in previous studies [13,14,15,16,34]. As urine can be collected non-invasively and is easily accessible, our study showed an inverse relationship between urinary Gal-3 levels and eGFR, and a positive relationship between creatinine and UPCR. In our study, patients in the highest tertile of urinary Gal-3 levels were associated with a 4.60-fold greater risk of kidney disease progression compared to those in the lowest tertile.

Numerous studies have found that the tubulointerstitial injury is more important in determining the subsequent decline in renal function than that caused by glomerular damage [35,36,37]. Following tubulointerstitial injury, inflammatory cells infiltrate in the interstitium, triggering inflammatory responses as well as the production of mediators involved in the fibrosis pathway, such as TGF-β [38,39]. Therefore, regardless of the underlying cause, tubulointerstitial fibrosis may be one of the most important pathogeneses of kidney disease progression [40,41]. This study found that higher levels of urinary Gal-3 were associated with global sclerosis, interstitial fibrosis and tubular atrophy. Based on the histopathological findings of our study, urinary Gal-3 is not only a urine biomarker for kidney disease progression but may also play a role in predicting renal fibrosis.

There have been several small studies that reported higher serum Gal-3 levels in diabetes patients compared with healthy controls, and Gal-3 was a predictor of diabetes even after adjusting for traditional risk factors [42,43,44]. In a large, multiethnic population of a Dallas Heart Study including 6586 study participants, plasma Gal-3 was associated with diabetes prevalence and the incident diabetes mellitus, possibly through the inflammatory pathway contributing to impaired insulin secretion and β-cell fibrosis [45]. Interestingly, our study found that patients in the highest urinary Gal-3 tertile had higher proportions of diabetes mellitus. In addition, the diagnosis of diabetic nephropathy was associated with higher urinary Gal-3 levels based on kidney biopsy specimens.

In our study, we selected several genes of CKD biomarkers that have been identified as CKD predictors through various pathophysiological mechanisms responsible for CKD initiation and progression [22,46]. B2M belongs to the major histocompatibility class I molecules, which are found on the surface of most nucleated cells, and the levels rise as CKD progression and ESRD [47]. Kidney injury molecule-1, encoded by HAVCR1, is a type 1 transmembrane protein that is undetectable in normal kidneys but is markedly upregulated after kidney damage [48]. Neutrophil gelatinase-associated lipocalin, encoded by LCN2, is a ubiquitous lipocalin iron-carrying protein, which is released by renal tubular epithelial cells in response to cell injury [49]. PTX3 is a member of the pentraxin superfamily, which is rapidly produced and released by resident and innate immunity cells in response to kidney injury, and PTX3 levels were observed to rise as eGFR decreased [50]. GDF15 was responsible for inflammatory pathways involved in regulating cell apoptosis, repair, and proliferation and was found to be elevated as CKD progression [51]. Interleukin-6, encoded by IL6, is a pleiotropic cytokine and was found to contribute to acute and chronic kidney injury and fibrosis [52]. After kidney stress injury or cell damage, renal tubular cells enter the G1 phase and cell-cycle arrest occurs accompanied by the accumulation of damaged DNA [53,54]. The levels of IGFBP7 and TIMP-2 are found to be elevated in G1 cell-cycle arrest during tubular cell stress and injury [55]. Most previous studies have focused on IGFBP7 and TIMP-2 for their role in acute kidney injury (AKI) prediction [56,57]. Of note, another study involving 50 patients undergoing cardiac surgery with cardiopulmonary bypass found that TIMP-2 and IGFBP7 are important determinants to predict patients’ recovery from AKI [24]. Beta trace protein (BTP), encoded by PTGDS, has been found to be filtered freely in the kidneys and highly correlated with measured GFR and residual kidney function. BTP has been found to be increased as CKD progresses [58,59]. TGFB1 promotes renal fibrosis by activating the Smad signaling pathway to transcriptionally regulate renal inflammation and fibrosis [60]. In our kidney biopsy specimens, we observed that intrarenal mRNA expressions of LGALS3 were most correlated with IGFBP7, TIMP-2 and PTGDS as well as TGFB1, suggesting that LGALS3 may be involved in renal dysfunction and promotes fibrotic processes after kidney stress and injury.

The strengths of this study are the completeness of data collection and the verification of the histopathological findings and intrarenal mRNA expression in a biopsy-proven cohort. In addition, this study prospectively examines the predictive ability of the urinary Gal-3 level for the risk of kidney disease progression during the study period. The study, however, has several limitations. First, we used eGFR values, rather than inulin or cystatin C measurement, to identify renal function, as eGFR values are more convenient to obtain and are available for patients in clinical practice. Second, as the eGFR was measured at the time of biopsy, AKI cannot be ruled out. However, the etiologies of renal diseases can also be identified by histopathological findings in our study. In addition, we conducted follow-up studies to assess changes in the eGFR, thereby allowing the detection of AKI. Third, our study was based on data collected from a renal biopsy cohort; further research should be conducted with a variety of patient populations. Finally, the urinary Gal-3 level was measured once, at the time of kidney biopsy; studies of serial changes in urinary biomarkers may be warranted.

## 5. Conclusions

In our study, we found that the highest urinary Gal-3 tertile was associated with more severe renal fibrosis and combining urinary and plasma Gal-3 may provide the better predictive performance for kidney disease progression. The generalizability of our findings needs to be examined, and the findings need to be validated in additional studies. Further research is also needed to determine whether therapeutic measures targeting Gal-3 might delay the progression of CKD.

## Figures and Tables

**Table 1:** Clinical characteristics of the study participants stratified by tertiles of urinary Gal-3.

		Tertiles of Urinary Gal-3			
	All Patients	Lowest Tertile (<354.6 pg/mL)	Middle Tertile (354.6–510.7 pg/mL)	Highest Tertile (≥510.8 pg/mL)	*p* Value
	(*n* = 280)	(*n* = 93)	(*n* = 93)	(*n* = 94)	
Age, years	56.2 ± 16.5	54.7 ± 17.4	56.5 ± 17.3	57.3 ± 14.7	0.541
Male sex, *n* (%)	170 (60.7)	56 (60.2)	54 (58.1)	60 (63.8)	0.717
eGFR, mL/min/1.73 m^2^	46.8 ± 36.0	61.0 ± 36.0	51.0 ± 37.5	28.7 ± 25.7	<0.001 ^a^
≥60, mL/min/1.73 m^2^, *n* (%)	84 (30.0)	44 (47.3)	30 (32.3)	10 (10.6)	
<60, mL/min/1.73 m^2^, *n* (%)	196 (70.0)	49 (52.7)	63 (67.7)	84 (89.4)	
UPCR, g/g	5.6 ± 9.4	2.9 ± 4.2	4.6 ± 5.5	9.2 ± 14.0	<0.001 ^b^
Uric acid, mg/dL	6.7 ± 2.2	6.4 ± 2.2	6.6 ± 2.2	7.0 ± 2.2	0.298
Albumin, mg/dL	3.2 ± 0.9	3.4 ± 0.9	3.1 ± 0.8	3.1 ± 0.8	0.092
Alanine transaminase, U/L	19.4 ± 14.2	19.7 ± 12.2	19.0 ± 13.2	19.6 ± 17.0	0.944
Hypertension, *n* (%)	115 (41.1)	35 (37.6)	39 (41.9)	41 (43.6)	0.693
Dyslipidemia, *n* (%)	55 (19.6)	14 (15.1)	18 (19.4)	23 (24.5)	0.268
Diabetes mellitus, *n* (%)	73 (26.1)	17 (18.3)	23 (24.7)	33 (35.1)	0.030 ^c^
SLE, *n* (%)	11 (3.9)	4 (4.3)	5 (5.4)	2 (2.1)	0.507
Coronary artery disease, *n* (%)	23 (8.2)	8 (8.6)	7 (7.5)	8 (8.5)	0.957
Congestive heart failure, *n* (%)	50 (17.9)	15 (16.1)	14 (15.1)	21 (22.3)	0.372
Stroke, *n* (%)	12 (4.3)	7 (7.5)	1 (1.1)	4 (4.3)	0.094
COPD, *n* (%)	8 (2.9)	2 (2.2)	1 (1.1)	5 (5.3)	0.194
Peptic ulcer disease, *n* (%)	21 (7.5)	8 (8.6)	7 (7.5)	6 (6.4)	0.847
Malignancy, *n* (%)	64 (22.9)	18 (19.4)	18 (19.4)	28 (29.8)	0.146

^a^ Post hoc test of eGFR showed a significant difference between lowest vs. highest tertile (*p* < 0.001) and middle vs. highest tertile (*p* < 0.001). ^b^ Post hoc test of UPCR showed a significant difference between lowest vs. highest tertile (*p* < 0.001) and middle vs. highest tertile (*p* = 0.002). ^c^ Post hoc test of diabetes mellitus showed a significant difference between lowest vs. highest tertile (*p* = 0.024). Abbreviations: Gal-3, galectin-3; eGFR, estimated glomerular filtration rate; UPCR, urine protein–creatinine ratio; SLE, systemic lupus erythematosus; COPD, chronic obstructive pulmonary disease.

**Table 2:** Risks of kidney disease progression and urinary Gal-3 levels.

	Kidney Disease Progression *					
	Crude HR	95% CI	*p* Value	Adjusted HR ^†^	95% CI	*p* Value
Tertiles of urinary Gal-3 levels						
Highest vs. lowest tertile	5.33	3.33–8.87	<0.001	4.60	2.85–7.71	<0.001
Middle vs. lowest tertile	1.96	1.16–3.37	0.013	1.82	1.08–3.15	0.027
Continuous values of urinary Gal-3 levels (per 100 pg/mL)						
Urinary Gal-3 levels	1.22	1.16–1.27	<0.001	1.19	1.13–1.25	<0.001

* Kidney disease progression was defined as ≥40% decline in the eGFR from baseline or ESRD (i.e., the initiation of hemodialysis or peritoneal dialysis, the receipt of a kidney transplant or eGFR < 15 mL/min/1.73 m^2^). ^†^ Adjusted for the presence of diabetic nephropathy, glomerulonephritis or other pathological findings in kidney biopsy. Abbreviation: Gal-3, galectin-3; HR, hazard ratio; CI, confidence interval.

**Table 3:** NRI, IDI, AIC, BIC and adjusted R^2^ for the combined assessment of eGFR and Gal-3 in predicting kidney disease progression.

	**NRI and IDI Model Comparison**							
	**NRI**	**95% CI**		***p* Value ^†^**	**IDI**	**95% CI**		***p* Value ^†^**
Model 1: eGFR	-	-		-	-	-		-
Model 2: eGFR + Urinary Gal-3	0.5395	0.3133–0.7657		<0.001	0.0626	0.0344–0.0907		<0.001
Model 3: eGFR + Plasma Gal-3	0.7333	0.5172–0.9494		<0.001	0.0633	0.0333–0.0934		<0.001
Model 4: eGFR + Urinary Gal-3 + Plasma Gal-3	0.6040	0.3805–0.8277		<0.001	0.0909	0.0564–0.1254		<0.001
	**AIC and BIC and R Square Model Comparison**							
	**AIC**		**BIC**		**Adjusted R^2^**		***p* Value**	
Model 1: eGFR	287.6032		298.5076		0.3541		<0.001	
Model 2: eGFR + Urinary Gal-3	269.3027		283.8419		0.3971		<0.001	
Model 3: eGFR + Plasma Gal-3	267.0637		281.6029		0.4019		<0.001	
Model 4: eGFR + Urinary Gal-3 + Plasma Gal-3	264.8257		282.9997		0.4088		<0.001	

^†^*p* value vs. model 1. Abbreviations: NRI, net reclassification improvement; IDI, integrated discrimination improvement; AIC, Akaike information; BIC, Bayesian information criterion; eGFR, estimated glomerular filtration rate; Gal-3, galectin-3; CI, confidence interval.

**Table 4:** Logistic regression for urinary Gal-3 (per 100 pg/mL) and renal pathological findings.

Pathological Findings	β	SE	Odds Ratio	95% CI	*p* Value
Glomerular description					
Global sclerosis	0.137	0.071	1.147	1.008–1.328	0.052
Segmental sclerosis	0.046	0.051	1.048	0.947–1.163	0.365
GBM double contour *	0.237	0.065	1.268	1.123–1.447	<0.001
GBM rigid *	0.258	0.067	1.295	1.143–1.484	<0.001
GBM thickening *	0.220	0.064	1.246	1.106–1.420	0.001
GBM collapse *	0.154	0.058	1.166	1.046–1.315	0.009
GBM attenuation	0.079	0.053	1.082	0.978–1.206	0.136
Glomerular necrosis	−0.810	0.537	0.445	0.128–1.076	0.131
Glomerular inflammatory change	−0.045	0.066	0.956	0.844–1.102	0.494
Glomerular ischemic change	0.105	0.069	1.111	0.957–1.275	0.130
Endocapillary hypertrophy *	0.235	0.064	1.265	1.122–1.444	<0.001
Extracapillary hypertrophy	0.075	0.053	1.077	0.974–1.200	0.157
Tubulointerstital descriptions					
Tubulitis	0.063	0.095	1.065	0.905–1.305	0.505
Interstitial edema	0.123	0.111	1.131	0.838–1.371	0.266
Interstitial inflammation *	0.207	0.069	1.229	1.083–1.417	0.003
Interstitial fibrosis *	0.196	0.066	1.217	1.076–1.394	0.003
Tubular atrophy *	0.208	0.068	1.231	1.086–1.416	0.002
Acute tubular necrosis	0.045	0.107	1.046	0.807–1.247	0.674
Casts	0.130	0.522	1.139	0.692–4.131	0.804
Vasculature descriptions					
Hyaline arteriosclerosis	0.029	0.087	1.029	0.845–1.196	0.740
Vascular intimal fibrosis	−0.031	0.090	0.969	0.796–1.131	0.728
Vascular necrosis	−1.457	0.998	0.233	0.015–1.054	0.144

* Statistically significant and *p* value are shown in bold. Abbreviations: Gal-3, galectin-3; SE, standard error; CI, confidence interval; GBM, glomerular basement membrane.

## Data Availability

Data Availability Statement: Restrictions apply to the availability of these data. Interested groups should contact Shuo-Ming Ou at okokyytt@gmail.com to discuss access permission. Accessed date: 1 March 2022.

## Supplementary Materials

The following supporting information can be downloaded at: https://www.mdpi.com/article/10.3390/biomedicines10030585/s1, Table S1. The detailed pathological diagnoses of the study participants. Table S2. Clinical characteristics of the participants whose intrarenal mRNA expression was analyzed. Figure S1. The associations between different pathological diagnoses and estimated glomerular filtration rate. Figure S2. Correlation plots of intrarenal LGALS3 with (A) B2M, (B) HAVCR1, (C) LCN2, (D) PTX3, (E) IL6, (F) GDF15, (G) ACTA2, (H) DES and (I) EGF in kidney biopsy specimens.

## References

[1] Cockwell P., Fisher L.A. (2020). The global burden of chronic kidney disease. Lancet.

[2] Parmar M.S. (2002). Chronic renal disease. BMJ.

[3] Chen T.K., Knicely D.H., Grams M.E. (2019). Chronic Kidney Disease Diagnosis and Management: A Review. JAMA.

[4] Harpole M., Davis J., Espina V. (2016). Current state of the art for enhancing urine biomarker discovery. Expert Rev. Proteom..

[5] Thomas C.E., Sexton W., Benson K., Sutphen R., Koomen J. (2010). Urine collection and processing for protein biomarker discovery and quantification. Cancer Epidemiol. Prev. Biomark..

[6] Zandi-Nejad K., Eddy A.A., Glassock R.J., Brenner B.M. (2004). Why is proteinuria an ominous biomarker of progressive kidney disease?. Kidney Int..

[7] Song L., Tang J.W., Owusu L., Sun M.Z., Wu J., Zhang J. (2014). Galectin-3 in cancer. Clin. Chim. Acta.

[8] Funasaka T., Raz A., Nangia-Makker P. (2014). Galectin-3 in angiogenesis and metastasis. Glycobiology.

[9] Henderson N.C., Sethi T. (2009). The regulation of inflammation by galectin-3. Immunol. Rev..

[10] Li L.C., Li J., Gao J. (2014). Functions of galectin-3 and its role in fibrotic diseases. J. Pharmacol. Exp. Ther..

[11] Mackinnon A.C., Gibbons M.A., Farnworth S.L., Leffler H., Nilsson U.J., Delaine T., Simpson A.J., Forbes S.J., Hirani N., Gauldie J. (2012). Regulation of transforming growth factor-β1-driven lung fibrosis by galectin-3. Am. J. Respir. Crit. Care Med..

[12] Calvier L., Martinez-Martinez E., Miana M., Cachofeiro V., Rousseau E., Sádaba J.R., Zannad F., Rossignol P., López-Andrés N. (2015). The impact of galectin-3 inhibition on aldosterone-induced cardiac and renal injuries. JACC Heart Fail..

[13] O’Seaghdha C.M., Hwang S.J., Ho J.E., Vasan R.S., Levy D., Fox C.S. (2013). Elevated galectin-3 precedes the development of CKD. J. Am. Soc. Nephrol..

[14] Tang W.H., Shrestha K., Shao Z., Borowski A.G., Troughton R.W., Thomas J.D., Klein A.L. (2011). Usefulness of plasma galectin-3 levels in systolic heart failure to predict renal insufficiency and survival. Am. J. Cardiol..

[15] Sotomayor C.G., Te Velde-Keyzer C.A., Diepstra A., van Londen M., Pol R.A., Post A., Gans R.O.B., Nolte I.M., Slart R., de Borst M.H. (2021). Galectin-3 and Risk of Late Graft Failure in Kidney Transplant Recipients: A 10-year Prospective Cohort Study. Transplantation.

[16] Ou S.M., Tsai M.T., Chen H.Y., Li F.A., Tseng W.C., Lee K.H., Chang F.P., Lin Y.P., Yang R.B., Tarng D.C. (2021). Identification of Galectin-3 as Potential Biomarkers for Renal Fibrosis by RNA-Sequencing and Clinicopathologic Findings of Kidney Biopsy. Front. Med..

[17] Levey A.S., Stevens L.A., Schmid C.H., Zhang Y.L., Castro A.F., Feldman H.I., Kusek J.W., Eggers P., Van Lente F., Greene T. (2009). A new equation to estimate glomerular filtration rate. Ann. Intern. Med..

[18] Maksimowski N.A., Song X., Bae E.H., Reich H., John R., Pei Y., Scholey J.W., Nephrotic Syndrome Study Network (2021). Follistatin-Like-1 (FSTL1) Is a Fibroblast-Derived Growth Factor That Contributes to Progression of Chronic Kidney Disease. Int. J. Mol. Sci..

[19] Perkovic V., Koitka-Weber A., Cooper M.E., Schernthaner G., Pfarr E., Woerle H.J., von Eynatten M., Wanner C. (2020). Choice of endpoint in kidney outcome trials: Considerations from the EMPA-REG OUTCOME^®^ trial. Nephrol. Dial. Transplant..

[20] Tsai M.T., Tseng W.C., Ou S.M., Lee K.H., Yang C.Y., Tarng D.C. (2021). Comparison of Simplified Creatinine Index and Systemic Inflammatory Markers for Nutritional Evaluation of Hemodialysis Patients. Nutrients.

[21] Ko T.M., Kuo H.C., Chang J.S., Chen S.P., Liu Y.M., Chen H.W., Tsai F.J., Lee Y.C., Chen C.H., Wu J.Y. (2015). CXCL10/IP-10 is a biomarker and mediator for Kawasaki disease. Circ. Res..

[22] Lousa I., Reis F., Beirão I., Alves R., Belo L., Santos-Silva A. (2020). New Potential Biomarkers for Chronic Kidney Disease Management-A Review of the Literature. Int. J. Mol. Sci..

[23] Zehra M., Curry J.C., Pillai S.S., Lakhani H.V., Edwards C.E., Sodhi K. (2020). Elucidating Potential Profibrotic Mechanisms of Emerging Biomarkers for Early Prognosis of Hepatic Fibrosis. Int. J. Mol. Sci..

[24] Meersch M., Schmidt C., Van Aken H., Martens S., Rossaint J., Singbartl K., Görlich D., Kellum J.A., Zarbock A. (2014). Urinary TIMP-2 and IGFBP7 as early biomarkers of acute kidney injury and renal recovery following cardiac surgery. PLoS ONE.

[25] Vijayan A., Faubel S., Askenazi D.J., Cerda J., Fissell W.H., Heung M., Humphreys B.D., Koyner J.L., Liu K.D., Mour G. (2016). Clinical Use of the Urine Biomarker [TIMP-2] × [IGFBP7] for Acute Kidney Injury Risk Assessment. Am. J. Kidney Dis..

[26] Jun J.I., Lau L.F. (2018). Resolution of organ fibrosis. J. Clin. Investig..

[27] Cook N.R., Ridker P.M. (2009). Advances in measuring the effect of individual predictors of cardiovascular risk: The role of reclassification measures. Ann. Intern. Med..

[28] Pencina M.J., D’Agostino R.B., Demler O.V. (2012). Novel metrics for evaluating improvement in discrimination: Net reclassification and integrated discrimination improvement for normal variables and nested models. Stat. Med..

[29] Dziak J.J., Coffman D.L., Lanza S.T., Li R., Jermiin L.S. (2020). Sensitivity and specificity of information criteria. Brief. Bioinform..

[30] Rozet E., Ziemons E., Marini R.D., Hubert P. (2013). Usefulness of information criteria for the selection of calibration curves. Anal. Chem..

[31] Ennis J.L., Luo D., Asplin J.R., Coe F.L. (2019). A laboratory-based algorithm to predict future kidney function decline in older adults with reduced estimated glomerular filtration rate. Clin. Nephrol..

[32] Herath N., Dassanayake R., Dissanayake M., Janitha C., Weerakoon K., Kumarasinghe C., de Silva T.G., Agampodi S. (2019). Normality data of eGFR and validity of commonly used screening tests for CKD in an area with endemic CKD of unknown etiology; need for age and sex based precise cutoff values. BMC Nephrol..

[33] Johnson D.W., Jones G.R., Mathew T.H., Ludlow M.J., Chadban S.J., Usherwood T., Polkinghorne K., Colagiuri S., Jerums G., Macisaac R. (2012). Chronic kidney disease and measurement of albuminuria or proteinuria: A position statement. Med. J. Aust..

[34] Rebholz C.M., Selvin E., Liang M., Ballantyne C.M., Hoogeveen R.C., Aguilar D., McEvoy J.W., Grams M.E., Coresh J. (2018). Plasma galectin-3 levels are associated with the risk of incident chronic kidney disease. Kidney Int..

[35] Risdon R.A., Sloper J.C., De Wardener H.E. (1968). Relationship between renal function and histological changes found in renal-biopsy specimens from patients with persistent glomerular nephritis. Lancet.

[36] Schainuck L.I., Striker G.E., Cutler R.E., Benditt E.P. (1970). Structural-functional correlations in renal disease. II. The correlations. Hum. Pathol..

[37] Striker G.E., Schainuck L.I., Cutler R.E., Benditt E.P. (1970). Structural-functional correlations in renal disease. I. A method for assaying and classifying histopathologic changes in renal disease. Hum. Pathol..

[38] Meng X.M., Nikolic-Paterson D.J., Lan H.Y. (2014). Inflammatory processes in renal fibrosis. Nat. Rev. Nephrol..

[39] Black L.M., Lever J.M., Agarwal A. (2019). Renal Inflammation and Fibrosis: A Double-edged Sword. J. Histochem. Cytochem..

[40] Hewitson T.D., Holt S.G., Smith E.R. (2017). Progression of Tubulointerstitial Fibrosis and the Chronic Kidney Disease Phenotype—Role of Risk Factors and Epigenetics. Front. Pharmacol..

[41] Hodgkins K.S., Schnaper H.W. (2012). Tubulointerstitial injury and the progression of chronic kidney disease. Pediatric Nephrol..

[42] Weigert J., Neumeier M., Wanninger J., Bauer S., Farkas S., Scherer M.N., Schnitzbauer A., Schäffler A., Aslanidis C., Schölmerich J. (2010). Serum galectin-3 is elevated in obesity and negatively correlates with glycosylated hemoglobin in type 2 diabetes. J. Clin. Endocrinol. Metab..

[43] Ohkura T., Fujioka Y., Nakanishi R., Shiochi H., Sumi K., Yamamoto N., Matsuzawa K., Izawa S., Ohkura H., Ueta E. (2014). Low serum galectin-3 concentrations are associated with insulin resistance in patients with type 2 diabetes mellitus. Diabetol. Metab. Syndr..

[44] Yilmaz H., Cakmak M., Inan O., Darcin T., Akcay A. (2015). Increased levels of galectin-3 were associated with prediabetes and diabetes: New risk factor?. J. Endocrinol. Investig..

[45] Vora A., de Lemos J.A., Ayers C., Grodin J.L., Lingvay I. (2019). Association of Galectin-3 with Diabetes Mellitus in the Dallas Heart Study. J. Clin. Endocrinol. Metab..

[46] Lopez-Giacoman S., Madero M. (2015). Biomarkers in chronic kidney disease, from kidney function to kidney damage. World J. Nephrol..

[47] Stefanović V., Djukanović L., Cukuranović R., Bukvić D., Ležaić V., Marić I., Ogrizovic S.S., Jovanović I., Vlahovic P., Pešić I. (2011). Beta2-microglobulin and alpha1-microglobulin as markers of Balkan endemic nephropathy, a worldwide disease. Ren. Fail..

[48] Sabbisetti V.S., Waikar S.S., Antoine D.J., Smiles A., Wang C., Ravisankar A., Ito K., Sharma S., Ramadesikan S., Lee M. (2014). Blood kidney injury molecule-1 is a biomarker of acute and chronic kidney injury and predicts progression to ESRD in type I diabetes. J. Am. Soc. Nephrol..

[49] Bolignano D., Donato V., Coppolino G., Campo S., Buemi A., Lacquaniti A., Buemi M. (2008). Neutrophil gelatinase-associated lipocalin (NGAL) as a marker of kidney damage. Am. J. Kidney Dis..

[50] Sjöberg B., Qureshi A.R., Heimbürger O., Stenvinkel P., Lind L., Larsson A., Bárány P., Ärnlöv J. (2016). Association between levels of pentraxin 3 and incidence of chronic kidney disease in the elderly. J. Intern. Med..

[51] Nair V., Robinson-Cohen C., Smith M.R., Bellovich K.A., Bhat Z.Y., Bobadilla M., Brosius F., de Boer I.H., Essioux L., Formentini I. (2017). Growth Differentiation Factor-15 and Risk of CKD Progression. J. Am. Soc. Nephrol..

[52] Sanchez-Alamo B., Shabaka A., Cachofeiro V., Cases-Corona C., Fernandez-Juarez G. (2022). Serum interleukin-6 levels predict kidney disease progression in diabetic nephropathy. Clin. Nephrol..

[53] Yang Q.H., Liu D.W., Long Y., Liu H.Z., Chai W.Z., Wang X.T. (2009). Acute renal failure during sepsis: Potential role of cell cycle regulation. J. Infect..

[54] Rodier F., Campisi J., Bhaumik D. (2007). Two faces of p53: Aging and tumor suppression. Nucleic Acids Res..

[55] Peng Z.-Y., Zhou F., Kellum J.A. (2016). Cross-species validation of cell cycle arrest markers for acute kidney injury in the rat during sepsis. Intensive Care Med. Exp..

[56] Kashani K., Al-Khafaji A., Ardiles T., Artigas A., Bagshaw S.M., Bell M., Bihorac A., Birkhahn R., Cely C.M., Chawla L.S. (2013). Discovery and validation of cell cycle arrest biomarkers in human acute kidney injury. Crit. Care.

[57] Zhang D., Yuan Y., Guo L., Wang Q. (2019). Comparison of urinary TIMP-2 and IGFBP7 cut-offs to predict acute kidney injury in critically ill patients: A PRISMA-compliant systematic review and meta-analysis. Medicine.

[58] Li T., Wilcox C.S., Lipkowitz M.S., Gordon-Cappitelli J., Dragoi S. (2019). Rationale and Strategies for Preserving Residual Kidney Function in Dialysis Patients. Am. J. Nephrol..

[59] Donadio C., Bozzoli L. (2016). Urinary β-trace protein: A unique biomarker to screen early glomerular filtration rate impairment. Medicine.

[60] Meng X.M., Tang P.M., Li J., Lan H.Y. (2015). TGF-β/Smad signaling in renal fibrosis. Front. Physiol..

