# Supplementary material for: Urinary Galectin-3 as a Novel Biomarker for the Prediction of Renal Fibrosis and Kidney Disease Progression
Source: Biomedicines. 2022 Mar 2;10(3):585. doi: 10.3390/biomedicines10030585 (PMC8945118; doi:10.3390/biomedicines10030585)

Urinary Galectin-3 as a Novel Biomarker for the Prediction of Renal Fibrosis and Kidney Disease Progression

Authors: Shuo-Ming Ou, Ming-Tsun Tsai, Huan-Yuan Chen, Fu-An Li, Kuo-Hua Lee, Wei-Cheng Tseng, Fu-Pang Chang, Yao-Ping Lin, Ruey-Bing Yang and Der-Cherng Tarng

Contents

**Supplemental Tables**	
Table S1. The detailed pathological diagnoses of the study participants	2
Table S2. Clinical characteristics of the participants whose intrarenal mRNA expression was analyzed	4
**Supplemental Figure**	
Figure S1. The associations between different pathological diagnoses and estimated glomerular filtration rate	5
Figure S2. Correlation plots of intrarenal LGALS3 with (A)B2M, (B)HAVCR1, (C)LCN2, (D)PTX3, (E)IL6, (F)GDF15, (G)ACTA2, (H)DES and (I)EGF in kidney biopsy specimens	6

Table S1. The detailed pathological diagnoses of the study participants

Proliferative GN<br>*n* = 61	Nonproliferative GN<br>*n* = 75	DN<br>*n* = 53	Vascular<br>*n* = 25	Tubulointerstitial<br>*n* = 21	Advanced<br>*n* = 27	Paraprotein<br>*n* = 8	Others<br>*n* = 10
IgA nephropathy (34)<br>Lupus nephritis (6)<br>ANCA-associated vasculitis (5)<br>Immune complex GN (5)<br>MesPGN (5)<br>Fibrillary GN (2)<br>MPGN (2)<br>DDD (1)<br>C3 GN (1)	Membranous GN (32)<br>Minimal change disease (25)<br>FSGS (16)<br>Collapsing glomerulopathy (1)<br>Focal mesangiolysis (1)	DN (53)	TMA (8)<br>Hypertensive nephropathy (17)	ATN (7)<br>AIN (6)<br>CIN (5)<br>Oxalate nephropathy (2)<br>Xanthogranulomatous pyelonephritis (1)	Advanced chronic changes (22)<br>Secondary FSGS (5)	Amyloidosis (3)<br>Heavy chain cast nephropathy (2)<br>Light chain cast nephropathy (3)	Normal (4)<br>TBM (4)<br>Fabry disease (1)<br>Cancer infiltration (1)

Abbreviations: GN, glomerulonephritis; DN, diabetic nephropathy; IgA, immunoglobulin A; ANCA, anti-neutrophil cytoplasmic antibodies; MesPGN, Mesangial proliferative glomerulonephritis; MPGN, membranoproliferative glomerulonephritis; DDD, dense deposit disease; FSGS, focal segmental glomerulosclerosis; TMA, thrombotic microangiopathy; ATN, acute tubular necrosis; AIN, acute interstitial nephritis; CIN, chronic interstitial nephritis; TBM, thin basement membrane disease.

Table S2. Clinical characteristics of the participants whose intrarenal mRNA expression was analyzed

	**Patients (n = 50)**
**Age, years**	54.5 ± 16.7
**Male sex, *n*(%)**	28 (56.0)
**eGFR, mL/min/1.73m^2**	56.1 ± 38.2
**≥ 60, mL/min/1.73m^2, *n*(%)**	22 (44.0)
**<60, mL/min/1.73m^2, *n*(%)**	28 (56.0)
**UPCR, g/g**	5.0 ± 5.7
**Uric acid, mg/dL**	7.3 ± 2.6
**Albumin, mg/dL**	3.3 ± 0.9
**Alanine transaminase, U/L**	19.5 ± 11.6
**Hypertension, *n*(%)**	23 (46.0)
**Dyslipidemia, *n*(%)**	8 (16.0)
**Diabetes mellitus, *n*(%)**	12 (24.0)
**SLE, *n*(%)**	3 (6.0)
**Coronary artery disease, *n*(%)**	1 (2.0)
**Congestive heart failure, *n*(%)**	7 (14.0)
**Stroke, *n*(%)**	0 (0.0)
**COPD, *n*(%)**	3 (6.0)
**Peptic ulcer disease, *n*(%)**	2 (4.0)
**Malignancy, *n*(%)**	6 (12.0)

Abbreviations: eGFR, estimated glomerular filtration rate; UPCR, urine protein-creatinine ratio; SLE, systemic lupus erythematosus; COPD, chronic obstructive pulmonary disease.

Figure S1. The associations between different pathological diagnoses and estimated glomerular filtration rate

Abbreviations: eGFR, estimated glomerular filtration rate; MCD, minimal change disease; MGN, membranous glomerulonephritis; FSGS, focal segmental glomerulosclerosis; IgA, immunoglobulin A; DM, diabetes mellitus.

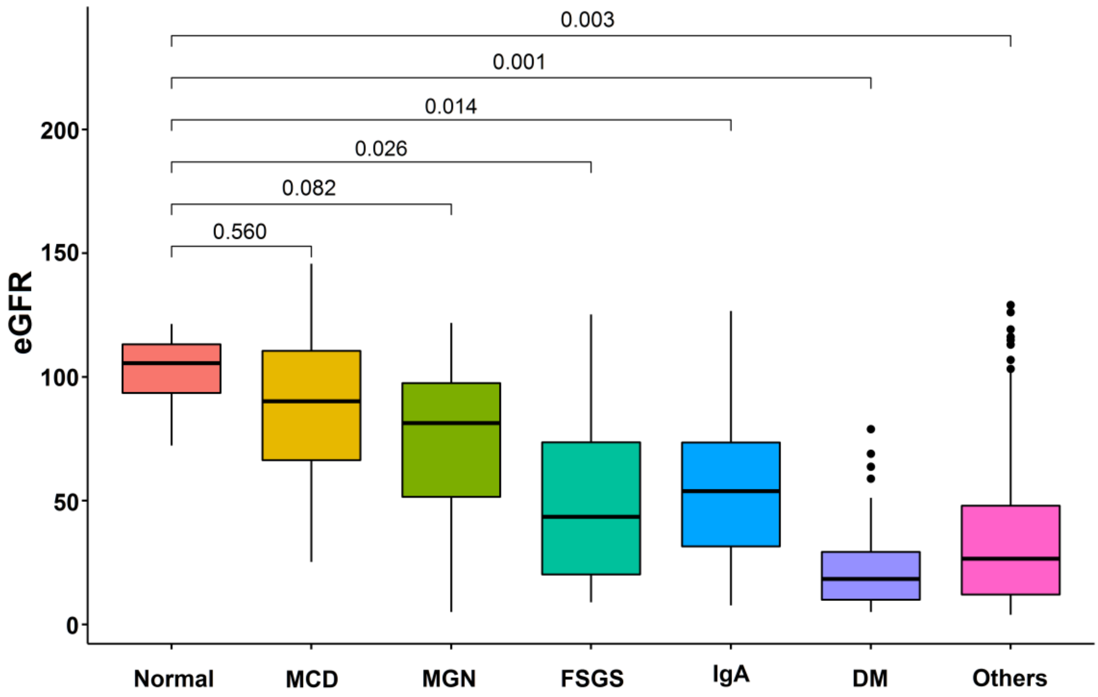

Figure S2. Correlation plots of intrarenal LGALS3 with (A)B2M, (B)HAVCR1, (C)LCN2, (D)PTX3, (E)IL6, (F)GDF15, (G)ACTA2, (H)DES and (I)EGF in kidney biopsy specimens.

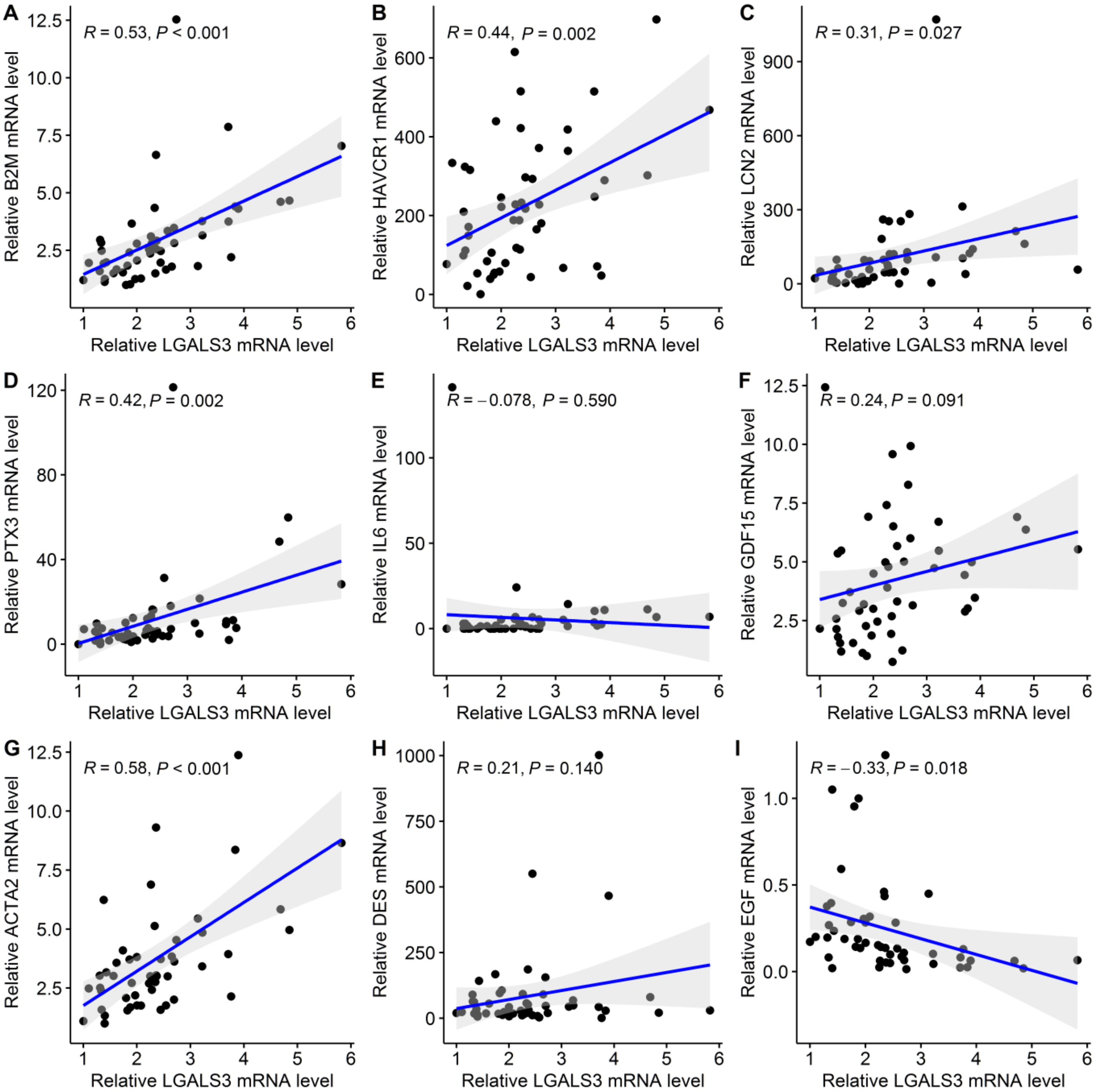

Supplement: Supplementary file 1 [file biomedicines-10-00585-s001.zip › biomedicines-1606483-supplementary.pdf]
